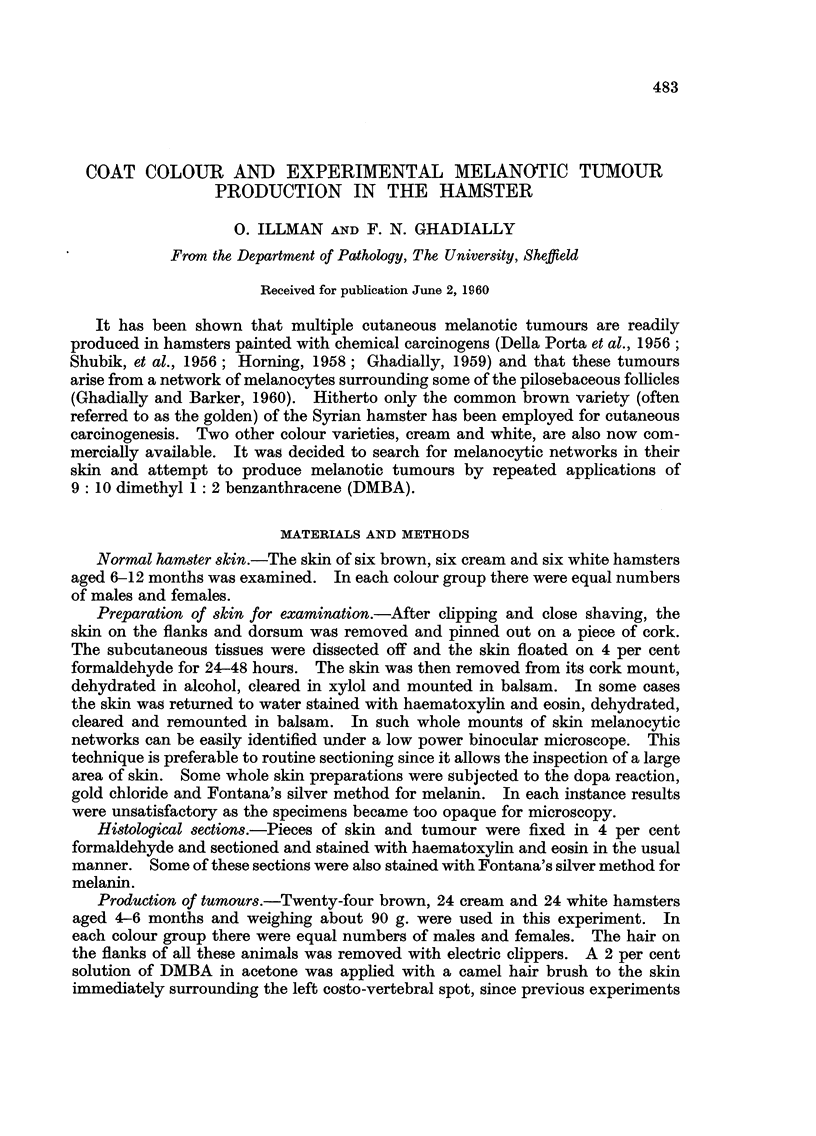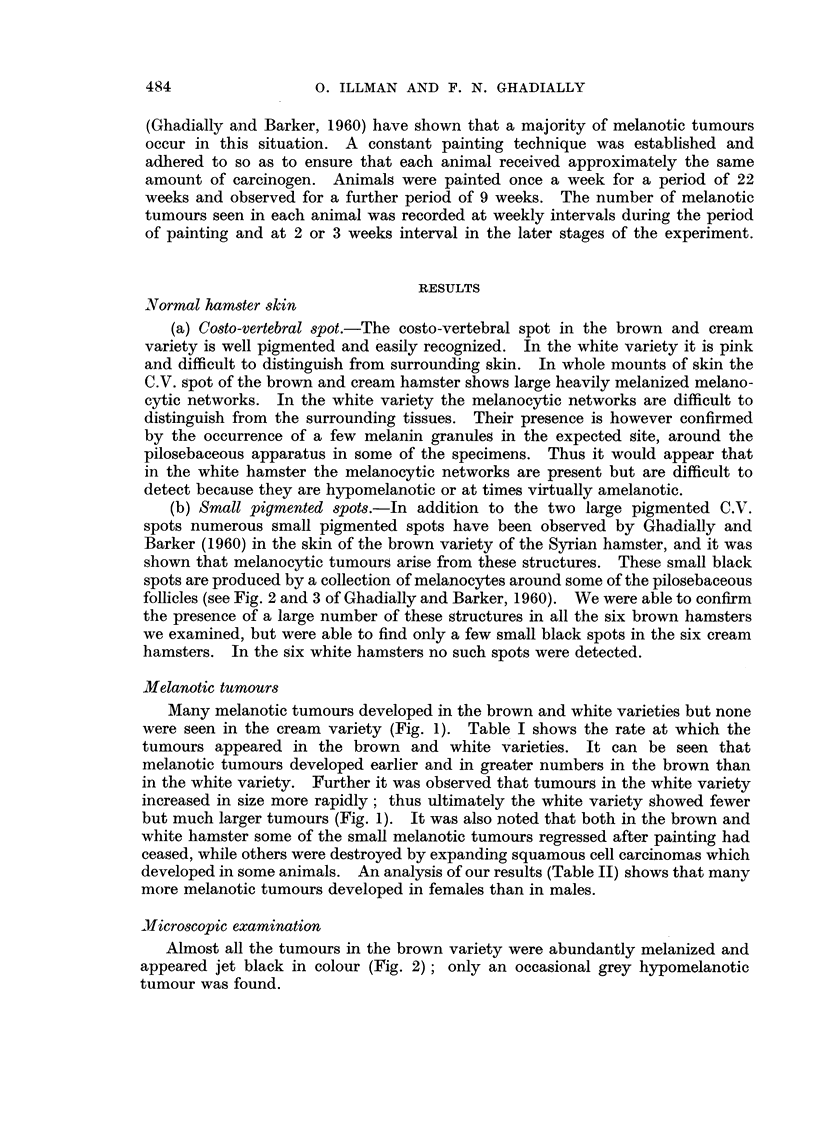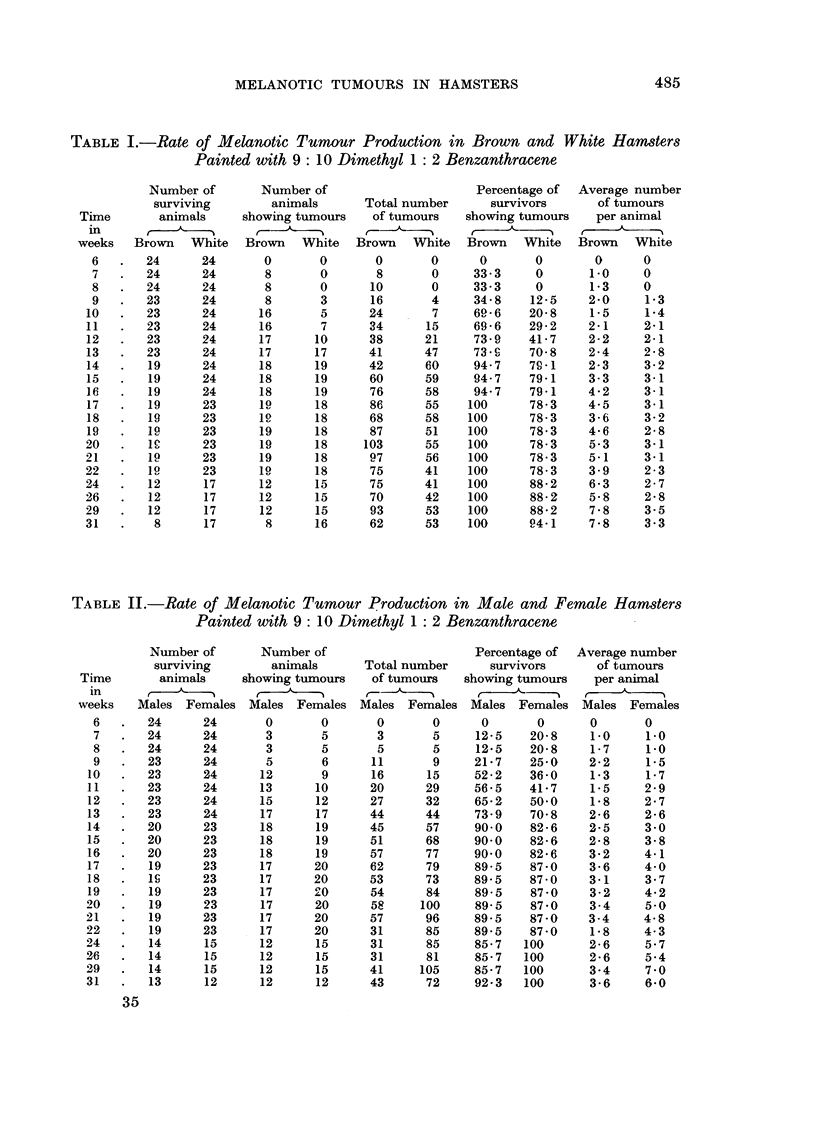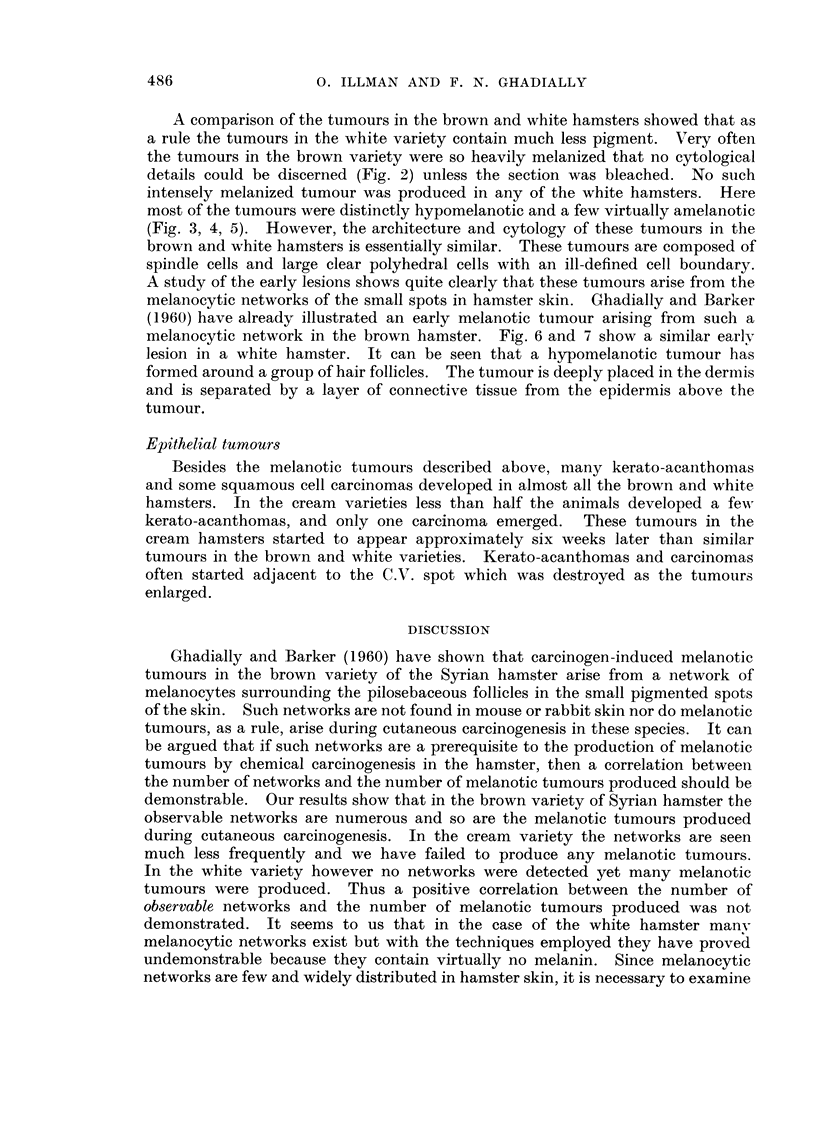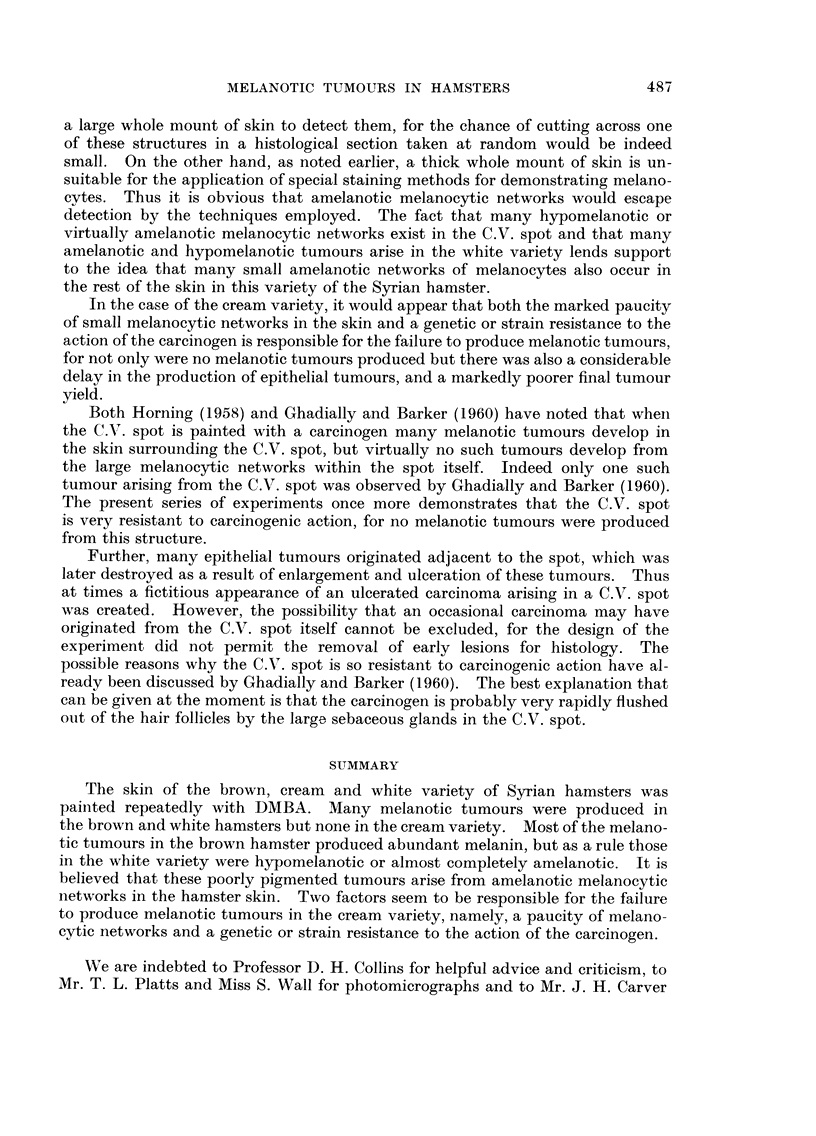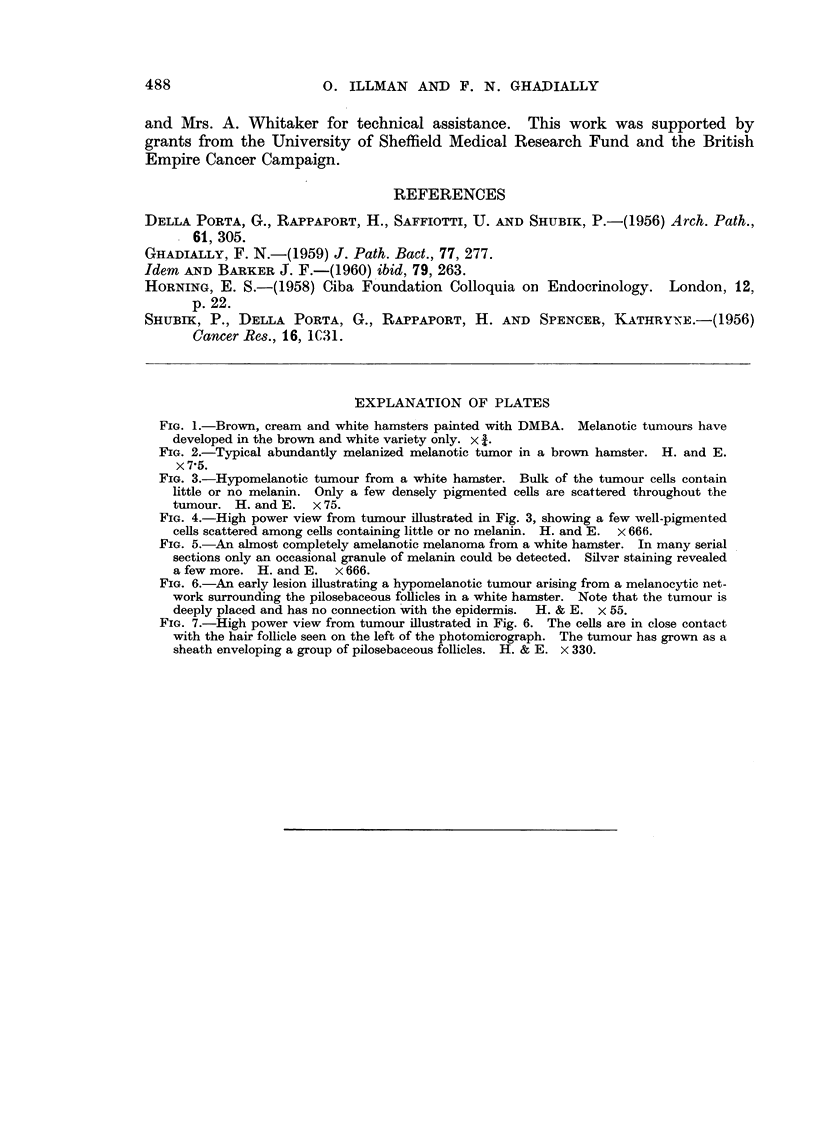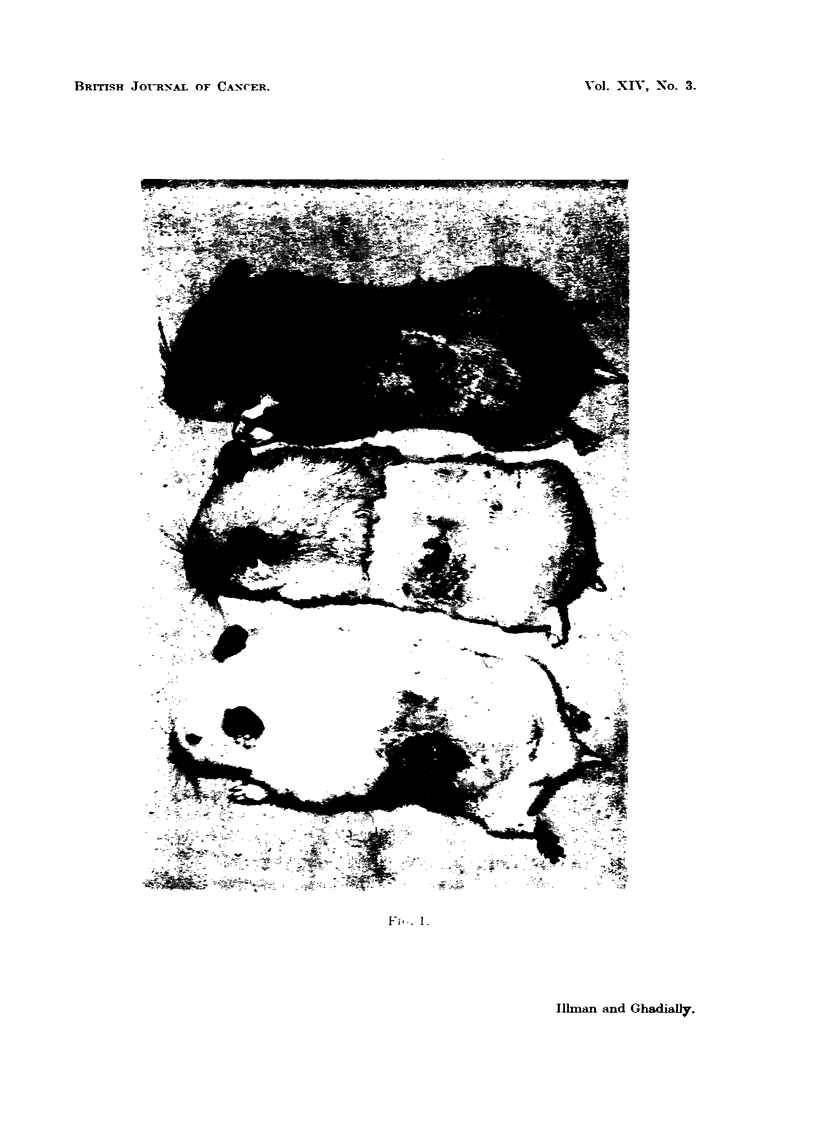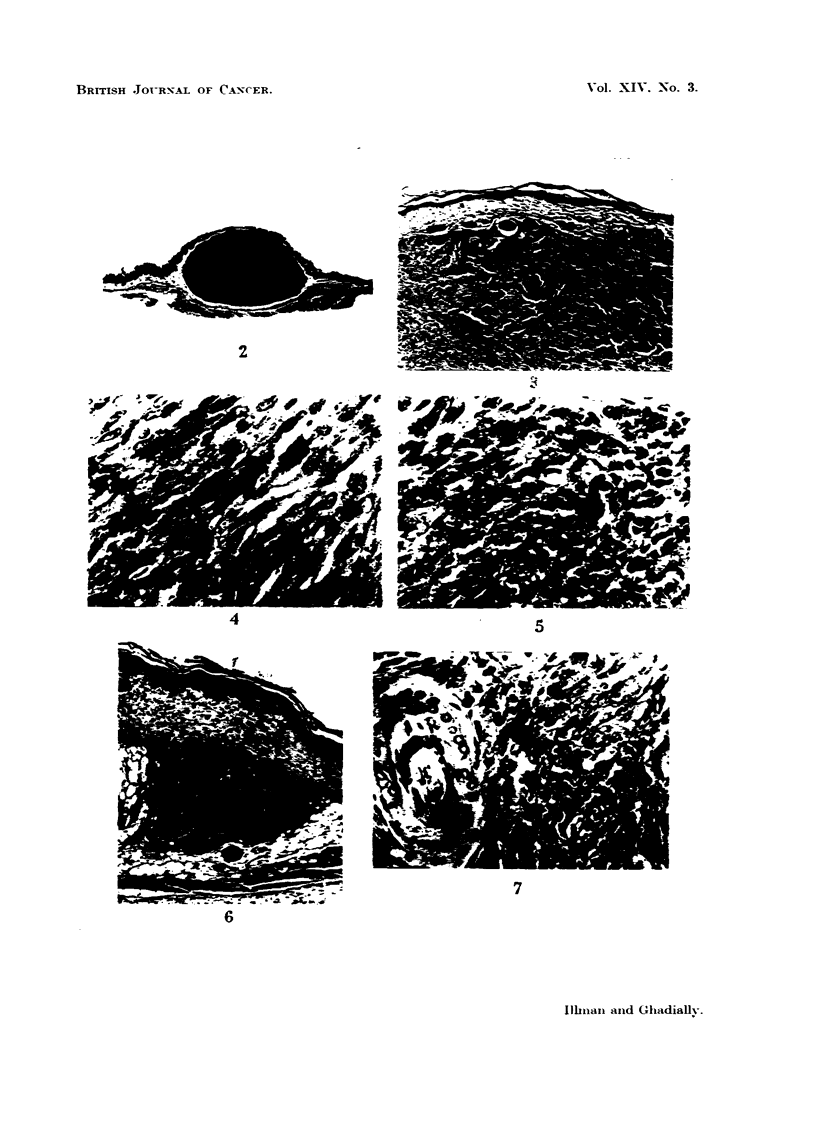# Coat Colour and Experimental Melanotic Tumour Production in the Hamster

**DOI:** 10.1038/bjc.1960.53

**Published:** 1960-09

**Authors:** O. Illman, F. N. Ghadially

## Abstract

**Images:**


### COAT COLOUR AND EXPERIMENTAL MELANOTIC TUMOUR PRODUCTION IN THE HAMSTER

O. ILLMAN AND F. N. GHADIALLY

*From the Department of Pathology, The University, Sheffield*

Received for publication June 2, 1960

It has been shown that multiple cutaneous melanotic tumours are readily produced in hamsters painted with chemical carcinogens (Della Porta *et al.*, 1956; Shubik, *et al.*, 1956; Horning, 1958; Ghadially, 1959) and that these tumours arise from a network of melanocytes surrounding some of the pilosebaceous follicles (Ghadially and Barker, 1960). Hitherto only the common brown variety (often referred to as the golden) of the Syrian hamster has been employed for cutaneous carcinogenesis. Two other colour varieties, cream and white, are also now commercially available. It was decided to search for melanocytic networks in their skin and attempt to produce melanotic tumours by repeated applications of 9 : 10 dimethyl 1 : 2 benzanthracene (DMBA).

### MATERIALS AND METHODS

*Normal hamster skin.*—The skin of six brown, six cream and six white hamsters aged 6–12 months was examined. In each colour group there were equal numbers of males and females.

*Preparation of skin for examination.*—After clipping and close shaving, the skin on the flanks and dorsum was removed and pinned out on a piece of cork. The subcutaneous tissues were dissected off and the skin floated on 4 per cent formaldehyde for 24–48 hours. The skin was then removed from its cork mount, dehydrated in alcohol, cleared in xylol and mounted in balsam. In some cases the skin was returned to water stained with haematoxylin and eosin, dehydrated, cleared and remounted in balsam. In such whole mounts of skin melanocytic networks can be easily identified under a low power binocular microscope. This technique is preferable to routine sectioning since it allows the inspection of a large area of skin. Some whole skin preparations were subjected to the dopa reaction, gold chloride and Fontana's silver method for melanin. In each instance results were unsatisfactory as the specimens became too opaque for microscopy.

*Histological sections.*—Pieces of skin and tumour were fixed in 4 per cent formaldehyde and sectioned and stained with haematoxylin and eosin in the usual manner. Some of these sections were also stained with Fontana's silver method for melanin.

*Production of tumours.*—Twenty-four brown, 24 cream and 24 white hamsters aged 4–6 months and weighing about 90 g. were used in this experiment. In each colour group there were equal numbers of males and females. The hair on the flanks of all these animals was removed with electric clippers. A 2 per cent solution of DMBA in acetone was applied with a camel hair brush to the skin immediately surrounding the left costo-vertebral spot, since previous experiments

(Ghadially and Barker, 1960) have shown that a majority of melanotic tumours occur in this situation. A constant painting technique was established and adhered to so as to ensure that each animal received approximately the same amount of carcinogen. Animals were painted once a week for a period of 22 weeks and observed for a further period of 9 weeks. The number of melanotic tumours seen in each animal was recorded at weekly intervals during the period of painting and at 2 or 3 weeks interval in the later stages of the experiment.

### RESULTS

### *Normal hamster skin*

(a) *Costo-vertebral spot.*—The costo-vertebral spot in the brown and cream variety is well pigmented and easily recognized. In the white variety it is pink and difficult to distinguish from surrounding skin. In whole mounts of skin the C.V. spot of the brown and cream hamster shows large heavily melanized melanocytic networks. In the white variety the melanocytic networks are difficult to distinguish from the surrounding tissues. Their presence is however confirmed by the occurrence of a few melanin granules in the expected site, around the pilosebaceous apparatus in some of the specimens. Thus it would appear that in the white hamster the melanocytic networks are present but are difficult to detect because they are hypomelanotic or at times virtually amelanotic.

(b) *Small pigmented spots.*—In addition to the two large pigmented C.V. spots numerous small pigmented spots have been observed by Ghadially and Barker (1960) in the skin of the brown variety of the Syrian hamster, and it was shown that melanocytic tumours arise from these structures. These small black spots are produced by a collection of melanocytes around some of the pilosebaceous follicles (see Fig. 2 and 3 of Ghadially and Barker, 1960). We were able to confirm the presence of a large number of these structures in all the six brown hamsters we examined, but were able to find only a few small black spots in the six cream hamsters. In the six white hamsters no such spots were detected.

### *Melanotic tumours*

Many melanotic tumours developed in the brown and white varieties but none were seen in the cream variety (Fig. 1). Table I shows the rate at which the tumours appeared in the brown and white varieties. It can be seen that melanotic tumours developed earlier and in greater numbers in the brown than in the white variety. Further it was observed that tumours in the white variety increased in size more rapidly ; thus ultimately the white variety showed fewer but much larger tumours (Fig. 1). It was also noted that both in the brown and white hamster some of the small melanotic tumours regressed after painting had ceased, while others were destroyed by expanding squamous cell carcinomas which developed in some animals. An analysis of our results (Table II) shows that many more melanotic tumours developed in females than in males.

### *Microscopic examination*

Almost all the tumours in the brown variety were abundantly melanized and appeared jet black in colour (Fig. 2) ; only an occasional grey hypomelanotic tumour was found.

TABLE I.—*Rate of Melanotic Tumour Production in Brown and White Hamsters Painted with* 9 : 10 *Dimethyl* 1 : 2 *Benzanthracene*

| Time in weeks | Number of surviving animals | | Number of animals showing tumours | | Total number of tumours | | Percentage of survivors showing tumours | | Average number of tumours per animal | |
|---|---|---|---|---|---|---|---|---|---|---|
| | Brown | White | Brown | White | Brown | White | Brown | White | Brown | White |
| 6 | 24 | 24 | 0 | 0 | 0 | 0 | 0 | 0 | 0 | 0 |
| 7 | 24 | 24 | 8 | 0 | 8 | 0 | 33·3 | 0 | 1·0 | 0 |
| 8 | 24 | 24 | 8 | 0 | 10 | 0 | 33·3 | 0 | 1·3 | 0 |
| 9 | 23 | 24 | 8 | 3 | 16 | 4 | 34·8 | 12·5 | 2·0 | 1·3 |
| 10 | 23 | 24 | 16 | 5 | 24 | 7 | 69·6 | 20·8 | 1·5 | 1·4 |
| 11 | 23 | 24 | 16 | 7 | 34 | 15 | 69·6 | 29·2 | 2·1 | 2·1 |
| 12 | 23 | 24 | 17 | 10 | 38 | 21 | 73·9 | 41·7 | 2·2 | 2·1 |
| 13 | 23 | 24 | 17 | 17 | 41 | 47 | 73·9 | 70·8 | 2·4 | 2·8 |
| 14 | 19 | 24 | 18 | 19 | 42 | 60 | 94·7 | 79·1 | 2·3 | 3·2 |
| 15 | 19 | 24 | 18 | 19 | 60 | 59 | 94·7 | 79·1 | 3·3 | 3·1 |
| 16 | 19 | 24 | 18 | 19 | 76 | 58 | 94·7 | 79·1 | 4·2 | 3·1 |
| 17 | 19 | 23 | 19 | 18 | 86 | 55 | 100 | 78·3 | 4·5 | 3·1 |
| 18 | 19 | 23 | 19 | 18 | 68 | 58 | 100 | 78·3 | 3·6 | 3·2 |
| 19 | 19 | 23 | 19 | 18 | 87 | 51 | 100 | 78·3 | 4·6 | 2·8 |
| 20 | 19 | 23 | 19 | 18 | 103 | 55 | 100 | 78·3 | 5·3 | 3·1 |
| 21 | 19 | 23 | 19 | 18 | 97 | 56 | 100 | 78·3 | 5·1 | 3·1 |
| 22 | 19 | 23 | 19 | 18 | 75 | 41 | 100 | 78·3 | 3·9 | 2·3 |
| 24 | 12 | 17 | 12 | 15 | 75 | 41 | 100 | 88·2 | 6·3 | 2·7 |
| 26 | 12 | 17 | 12 | 15 | 70 | 42 | 100 | 88·2 | 5·8 | 2·8 |
| 29 | 12 | 17 | 12 | 15 | 93 | 53 | 100 | 88·2 | 7·8 | 3·5 |
| 31 | 8 | 17 | 8 | 16 | 62 | 53 | 100 | 94·1 | 7·8 | 3·3 |

TABLE II.—*Rate of Melanotic Tumour Production in Male and Female Hamsters Painted with* 9 : 10 *Dimethyl* 1 : 2 *Benzanthracene*

| Time in weeks | Number of surviving animals | | Number of animals showing tumours | | Total number of tumours | | Percentage of survivors showing tumours | | Average number of tumours per animal | |
|---|---|---|---|---|---|---|---|---|---|---|
| | Males | Females | Males | Females | Males | Females | Males | Females | Males | Females |
| 6 | 24 | 24 | 0 | 0 | 0 | 0 | 0 | 0 | 0 | 0 |
| 7 | 24 | 24 | 3 | 5 | 3 | 5 | 12·5 | 20·8 | 1·0 | 1·0 |
| 8 | 24 | 24 | 3 | 5 | 5 | 5 | 12·5 | 20·8 | 1·7 | 1·0 |
| 9 | 23 | 24 | 5 | 6 | 11 | 9 | 21·7 | 25·0 | 2·2 | 1·5 |
| 10 | 23 | 24 | 12 | 9 | 16 | 15 | 52·2 | 36·0 | 1·3 | 1·7 |
| 11 | 23 | 24 | 13 | 10 | 20 | 29 | 56·5 | 41·7 | 1·5 | 2·9 |
| 12 | 23 | 24 | 15 | 12 | 27 | 32 | 65·2 | 50·0 | 1·8 | 2·7 |
| 13 | 23 | 24 | 17 | 17 | 44 | 44 | 73·9 | 70·8 | 2·6 | 2·6 |
| 14 | 20 | 23 | 18 | 19 | 45 | 57 | 90·0 | 82·6 | 2·5 | 3·0 |
| 15 | 20 | 23 | 18 | 19 | 51 | 68 | 90·0 | 82·6 | 2·8 | 3·8 |
| 16 | 20 | 23 | 18 | 19 | 57 | 77 | 90·0 | 82·6 | 3·2 | 4·1 |
| 17 | 19 | 23 | 17 | 20 | 62 | 79 | 89·5 | 87·0 | 3·6 | 4·0 |
| 18 | 19 | 23 | 17 | 20 | 53 | 73 | 89·5 | 87·0 | 3·1 | 3·7 |
| 19 | 19 | 23 | 17 | 20 | 54 | 84 | 89·5 | 87·0 | 3·2 | 4·2 |
| 20 | 19 | 23 | 17 | 20 | 58 | 100 | 89·5 | 87·0 | 3·4 | 5·0 |
| 21 | 19 | 23 | 17 | 20 | 57 | 96 | 89·5 | 87·0 | 3·4 | 4·8 |
| 22 | 19 | 23 | 17 | 20 | 31 | 85 | 89·5 | 87·0 | 1·8 | 4·3 |
| 24 | 14 | 15 | 12 | 15 | 31 | 85 | 85·7 | 100 | 2·6 | 5·7 |
| 26 | 14 | 15 | 12 | 15 | 31 | 81 | 85·7 | 100 | 2·6 | 5·4 |
| 29 | 14 | 15 | 12 | 15 | 41 | 105 | 85·7 | 100 | 3·4 | 7·0 |
| 31 | 13 | 12 | 12 | 12 | 43 | 72 | 92·3 | 100 | 3·6 | 6·0 |

A comparison of the tumours in the brown and white hamsters showed that as a rule the tumours in the white variety contain much less pigment. Very often the tumours in the brown variety were so heavily melanized that no cytological details could be discerned (Fig. 2) unless the section was bleached. No such intensely melanized tumour was produced in any of the white hamsters. Here most of the tumours were distinctly hypomelanotic and a few virtually amelanotic (Fig. 3, 4, 5). However, the architecture and cytology of these tumours in the brown and white hamsters is essentially similar. These tumours are composed of spindle cells and large clear polyhedral cells with an ill-defined cell boundary. A study of the early lesions shows quite clearly that these tumours arise from the melanocytic networks of the small spots in hamster skin. Ghadially and Barker (1960) have already illustrated an early melanotic tumour arising from such a melanocytic network in the brown hamster. Fig. 6 and 7 show a similar early lesion in a white hamster. It can be seen that a hypomelanotic tumour has formed around a group of hair follicles. The tumour is deeply placed in the dermis and is separated by a layer of connective tissue from the epidermis above the tumour.

*Epithelial tumours*

Besides the melanotic tumours described above, many kerato-acanthomas and some squamous cell carcinomas developed in almost all the brown and white hamsters. In the cream varieties less than half the animals developed a few kerato-acanthomas, and only one carcinoma emerged. These tumours in the cream hamsters started to appear approximately six weeks later than similar tumours in the brown and white varieties. Kerato-acanthomas and carcinomas often started adjacent to the C.V. spot which was destroyed as the tumours enlarged.

### DISCUSSION

Ghadially and Barker (1960) have shown that carcinogen-induced melanotic tumours in the brown variety of the Syrian hamster arise from a network of melanocytes surrounding the pilosebaceous follicles in the small pigmented spots of the skin. Such networks are not found in mouse or rabbit skin nor do melanotic tumours, as a rule, arise during cutaneous carcinogenesis in these species. It can be argued that if such networks are a prerequisite to the production of melanotic tumours by chemical carcinogenesis in the hamster, then a correlation between the number of networks and the number of melanotic tumours produced should be demonstrable. Our results show that in the brown variety of Syrian hamster the observable networks are numerous and so are the melanotic tumours produced during cutaneous carcinogenesis. In the cream variety the networks are seen much less frequently and we have failed to produce any melanotic tumours. In the white variety however no networks were detected yet many melanotic tumours were produced. Thus a positive correlation between the number of *observable* networks and the number of melanotic tumours produced was not demonstrated. It seems to us that in the case of the white hamster many melanocytic networks exist but with the techniques employed they have proved undemonstrable because they contain virtually no melanin. Since melanocytic networks are few and widely distributed in hamster skin, it is necessary to examine a large whole mount of skin to detect them, for the chance of cutting across one of these structures in a histological section taken at random would be indeed small. On the other hand, as noted earlier, a thick whole mount of skin is unsuitable for the application of special staining methods for demonstrating melanocytes. Thus it is obvious that amelanotic melanocytic networks would escape detection by the techniques employed. The fact that many hypomelanotic or virtually amelanotic melanocytic networks exist in the C.V. spot and that many amelanotic and hypomelanotic tumours arise in the white variety lends support to the idea that many small amelanotic networks of melanocytes also occur in the rest of the skin in this variety of the Syrian hamster.

In the case of the cream variety, it would appear that both the marked paucity of small melanocytic networks in the skin and a genetic or strain resistance to the action of the carcinogen is responsible for the failure to produce melanotic tumours, for not only were no melanotic tumours produced but there was also a considerable delay in the production of epithelial tumours, and a markedly poorer final tumour yield.

Both Horning (1958) and Ghadially and Barker (1960) have noted that when the C.V. spot is painted with a carcinogen many melanotic tumours develop in the skin surrounding the C.V. spot, but virtually no such tumours develop from the large melanocytic networks within the spot itself. Indeed only one such tumour arising from the C.V. spot was observed by Ghadially and Barker (1960). The present series of experiments once more demonstrates that the C.V. spot is very resistant to carcinogenic action, for no melanotic tumours were produced from this structure.

Further, many epithelial tumours originated adjacent to the spot, which was later destroyed as a result of enlargement and ulceration of these tumours. Thus at times a fictitious appearance of an ulcerated carcinoma arising in a C.V. spot was created. However, the possibility that an occasional carcinoma may have originated from the C.V. spot itself cannot be excluded, for the design of the experiment did not permit the removal of early lesions for histology. The possible reasons why the C.V. spot is so resistant to carcinogenic action have already been discussed by Ghadially and Barker (1960). The best explanation that can be given at the moment is that the carcinogen is probably very rapidly flushed out of the hair follicles by the large sebaceous glands in the C.V. spot.

### SUMMARY

The skin of the brown, cream and white variety of Syrian hamsters was painted repeatedly with DMBA. Many melanotic tumours were produced in the brown and white hamsters but none in the cream variety. Most of the melanotic tumours in the brown hamster produced abundant melanin, but as a rule those in the white variety were hypomelanotic or almost completely amelanotic. It is believed that these poorly pigmented tumours arise from amelanotic melanocytic networks in the hamster skin. Two factors seem to be responsible for the failure to produce melanotic tumours in the cream variety, namely, a paucity of melanocytic networks and a genetic or strain resistance to the action of the carcinogen.

We are indebted to Professor D. H. Collins for helpful advice and criticism, to Mr. T. L. Platts and Miss S. Wall for photomicrographs and to Mr. J. H. Carver and Mrs. A. Whitaker for technical assistance. This work was supported by grants from the University of Sheffield Medical Research Fund and the British Empire Cancer Campaign.

### REFERENCES

DELLA PORTA, G., RAPPAPORT, H., SAFFIOTTI, U. AND SHUBIK, P.—(1956) *Arch. Path.*, **61**, 305.

GHADIALLY, F. N.—(1959) *J. Path. Bact.*, **77**, 277.

*Idem* AND BARKER J. F.—(1960) *ibid*, **79**, 263.

HORNING, E. S.—(1958) Ciba Foundation Colloquia on Endocrinology. London, **12**, p. 22.

SHUBIK, P., DELLA PORTA, G., RAPPAPORT, H. AND SPENCER, KATHRYNE.—(1956) *Cancer Res.*, **16**, 1031.

---

### EXPLANATION OF PLATES

FIG. 1.—Brown, cream and white hamsters painted with DMBA. Melanotic tumours have developed in the brown and white variety only. $\times \frac{3}{4}$.

FIG. 2.—Typical abundantly melanized melanotic tumor in a brown hamster. H. and E. $\times 7 \cdot 5$.

FIG. 3.—Hypomelanotic tumour from a white hamster. Bulk of the tumour cells contain little or no melanin. Only a few densely pigmented cells are scattered throughout the tumour. H. and E. $\times 75$.

FIG. 4.—High power view from tumour illustrated in Fig. 3, showing a few well-pigmented cells scattered among cells containing little or no melanin. H. and E. $\times 666$.

FIG. 5.—An almost completely amelanotic melanoma from a white hamster. In many serial sections only an occasional granule of melanin could be detected. Silver staining revealed a few more. H. and E. $\times 666$.

FIG. 6.—An early lesion illustrating a hypomelanotic tumour arising from a melanocytic network surrounding the pilosebaceous follicles in a white hamster. Note that the tumour is deeply placed and has no connection with the epidermis. H. & E. $\times 55$.

FIG. 7.—High power view from tumour illustrated in Fig. 6. The cells are in close contact with the hair follicle seen on the left of the photomicrograph. The tumour has grown as a sheath enveloping a group of pilosebaceous follicles. H. & E. $\times 330$.

---

FIG. 1.

Illman and Ghadially.